# Correspondence

**Published:** 1876-02

**Authors:** D. S. Brainard

**Affiliations:** Zumbrota, Minn.


### Correspondence.

ZUMBROTA, MINN., Jan. 9th, 1876.

*Editors Buffalo Medical and Surgical Journal:*

DEAR SIRS:—I will report a case which I think you will deem sufficiently interesting for publication in your Journal. The report of said case has been neglected until the present time, on account of each physician interested in the case waiting for another to write a report.

Case of Tracheotomy for the removal of a piece of clay pipestem from the right bronchial tube.

On the 14th day of October, 1875, a little boy, six years of age, while playing with a piece of clay pipe-stem in his month, accidently allowed it to pass backwards into the Pharynx, and it entered the Larynx, dropping down the Trachia into the right bronchial tube.

Dr. McKinstry of this place was called, and used all the means at his command for the removal of the foreign substance. Dr. Hill, of Pine Island, and myself were also summoned, and we all agreed upon an operation as the only means of offering any relief to the boy. Therefore, we at once proceeded to perform the operation of Tracheotomy, and succeeded in removing from the right bronchial tube a piece of clay pipe-stem one and seven-eighths inches in length. The boy bore the operation well, and made a rapid recovery. Eight weeks after the accident he attended school, and all the scar remaining at the point of incission, is a slight line.

Very truly yours,

D. S. BRAINARD, M. D.

